# Analyzing Digital Evidence From a Telemental Health Platform to Assess Complex Psychological Responses to the COVID-19 Pandemic: Content Analysis of Text Messages

**DOI:** 10.2196/26190

**Published:** 2021-02-09

**Authors:** Thomas D Hull, Jacob Levine, Niels Bantilan, Angel N Desai, Maimuna S Majumder

**Affiliations:** 1 Talkspace New York, NY United States; 2 Department of Counseling and Clinical Psychology Teachers College Columbia University New York, NY United States; 3 Department of Internal Medicine University of California at Davis Davis, CA United States; 4 Computational Health Informatics Program Boston Children's Hospital Harvard Medical School Boston, MA United States

**Keywords:** digital phenotyping, COVID-19, telehealth, digital mental health, natural language processing, machine learning, mental health, phenotyping, burden, treatment, symptom

## Abstract

**Background:**

The novel COVID-19 disease has negatively impacted mortality, economic conditions, and mental health. These impacts are likely to continue after the COVID-19 pandemic ends. There are no methods for characterizing the mental health burden of the COVID-19 pandemic, and differentiating this burden from that of the prepandemic era. Accurate illness detection methods are critical for facilitating pandemic-related treatment and preventing the worsening of symptoms.

**Objective:**

We aimed to identify major themes and symptom clusters in the SMS text messages that patients send to therapists. We assessed patients who were seeking treatment for pandemic-related distress on Talkspace, which is a popular telemental health platform.

**Methods:**

We used a machine learning algorithm to identify patients’ pandemic-related concerns, based on their SMS text messages in a large, digital mental health service platform (ie, Talkspace). This platform uses natural language processing methods to analyze unstructured therapy transcript data, in parallel with brief clinical assessment methods for analyzing depression and anxiety symptoms.

**Results:**

Our results show a significant increase in the incidence of COVID-19–related intake anxiety symptoms (*P*<.001), but no significant differences in the incidence of intake depression symptoms (*P*=.79). During our transcript analyses, we identified terms that were related to 24 symptoms outside of those included in the diagnostic criteria for anxiety and depression.

**Conclusions:**

Our findings for Talkspace suggest that people who seek treatment during the pandemic experience more severe intake anxiety than they did before the COVID-19 outbreak. It is important to monitor the symptoms that we identified in this study and the symptoms of anxiety and depression, to fully understand the effects of the COVID-19 pandemic on mental health.

## Introduction

Since late 2019, the ongoing COVID-19 pandemic has proved to be extremely disruptive; the pandemic has resulted in high morbidity and mortality rates, as well as economic and mental health consequences. These issues pose a challenge for mental health services, as little is known about SARS-CoV-2 and the psychological impact that the COVID-19 crisis has on medical professionals, essential workers, unemployed individuals, and other people who engage in physical distancing. Early reports have suggested that mental health professionals face considerable challenges due to the lack of information and established guidelines for assessing and treating patients with COVID-19 [1]. Psychological sequelae to major events like the COVID-19 pandemic vary greatly [2-4]. This makes it difficult to assess the full range of symptoms that are potentially related to the COVID-19 crisis, and to track the course of COVID-19–related reactions over time. The use of comprehensive symptom checklists and exhaustive clinical interviews poses a considerable burden to clinicians and respondents alike. However, this burden can be avoided if the most common symptoms are known ahead of time.

A major obstacle to identifying the most relevant symptoms for screening is the amount of time it takes to amass clinical observations for a suitably large patient population. Large patient populations are needed for determining the full scope of patients’ reactions. It is difficult to differentiate peripheral symptoms from central and pathogenic symptoms [5]. It is also difficult to differentiate symptoms that are ordinarily reported by a diverse, treatment-seeking population from symptoms that are more closely associated with COVID-19–related concerns. However, there is a lack of this type of data for the ongoing COVID-19 crisis. Data that are generated by large telemental health services that remotely deliver care (ie, US and global telemental health services) may be helpful in describing the full complexity of patients’ clinical presentations. Text messages between patients and therapists (ie, text messages that are a part of intake and treatment procedures) offer the most useful data. These data are relatively unstructured compared to standardized symptom measures, but they offer the advantage of capturing patients’ experiences more comprehensively. This allows therapists to differentiate symptoms that are reported in conjunction with mentions of COVID-19, from symptoms that are reported by individuals who seek care for other reasons. Natural language processing (NLP) methods refer to a broad set of methods that have been designed to analyze unstructured textual data. These methods range from simple methods that search for specific words in a block of text, to more complex neural network models that extract the meaning of certain statements by analyzing the larger context of a text corpus.

The aim of this study was to identify major themes and symptom clusters in the text messages that patients send to therapists. We assessed patients who were seeking treatment for pandemic-related distress on Talkspace, which is a popular telemental health platform. To achieve our objective, we differentiated symptoms that were associated with the pandemic from symptoms that were only experienced by individuals who seek treatment, by investigating the relationships among words that were associated with mentions of COVID-19. This is impossible to do when only relying on structured symptom measures that do not specify whether symptoms relate to the pandemic or some other cause. Therefore, we used a multistep process that involved brief symptom measures to determine the relationship between the pandemic and common, self-reported anxiety and depression symptoms; identify patients with COVID-19-related concerns; and isolate words that highly correlate with mentions of SARS-CoV-2. We categorized these words by using a digital phenotyping process for determining the prevalence of clinical and nonclinical themes, to ultimately identify symptoms in diagnostic categories that are not reflected in structured measures for common anxiety and depression symptoms. Our study demonstrates that our method has strong face validity and high levels of clinical interpretability. Therefore, our method can potentially be used to inform decisions on structured measures for tracking responses to the pandemic over time.

## Methods

### Setting

Talkspace is a telemental health platform that enables licensed psychotherapists to deliver care through asynchronous, two-way messaging methods, including text messaging, audio messaging, and video messaging. Talkspace also allows psychotherapists to schedule live video sessions with patients within their regions of licensure. Studies have shown that Talkspace is acceptable and feasible for increasing patients’ access to care [6]. Talkspace has been used by over 2000 therapists who each serve an average of 15-20 patients at any given time (ie, throughout the United States and worldwide). This platform allows for the seamless transfer of symptom and outcome measure data to therapists, and offers crisis and referral services to patients who need a higher level of care than what the messaging platform can offer.

### Participants

To evaluate changes in patients’ self-reported symptoms, we administered the 7-item Generalized Anxiety Disorder questionnaire (GAD-7) [7] and the 9-item Patient Health Questionnaire [8] to patients who started treatment for depression and anxiety between January 1, 2017 and June 9, 2020. This allowed us to compare pre–COVID-19 pandemic trends in self-reported symptoms against trends that are contemporaneous to the ongoing COVID-19 pandemic.

To evaluate symptoms that are not included in the standard measures for depression and anxiety, regular expressions that were related to the pandemic, including “corona,” “virus,” “covid,” and “pandemic,” were identified and assessed. We verified that all regular expressions had a near 0% incidence rate prior to February 2020 (see Table 1). We conducted computerized keyword matching to analyze and identify COVID-19–related terms from all patient messages in treatment transcripts that were generated between March 1, 2020 and June 9, 2020. In this study, we defined “transcript” as the set of all messages that were exchanged between a patient and a care provider. Therefore, each patient had exactly 1 transcript.

**Table 1:** Percentage of messages that contained pandemic-related seed words/regular expressions before and after the COVID-19 pandemic.

Month and year	% of messages that contained “corona”	% of messages that contained “virus”	% of messages that contained “covid”	% of messages that contained “pandemic”
January 2020	0.0182%	0.0683%	0%	0.0017%
February 2020	0.0785%	0.1604%	0.0045%	0.0074%
March 2020	1.1113%	2.2173%	0.9072%	0.6105%
April 2020	0.4743%	1.0913%	1.2988%	1.0372%
May 2020	0.2848%	0.5751%	1.1225%	0.9988%
June 2020	0.2688%	0.4727%	1.1481%	0.9735%

### Statistical Analysis

We calculated and aggregated average summary scores for patients’ anxiety and depression scale scores. Furthermore, we stratified these scores based on patients’ days of admission to assess changes over time. Statistical analyses were conducted with statistical analysis packages that use the Python programming language. Pandas was used for data analysis [9,10], Matplotlib [11] and Seaborn [12] were used for visualization, and Scipy [13] and Pandera [14] were used for data validation and hypothesis testing.

In order to identify the words and phrases (ie, n-grams) that are the most likely to appear with COVID-19–related mentions, we used NLP methods to represent each text day (ie, the days that text messages were sent) as a vector of word counts. These vectors were then transformed into term frequency-inverse document frequency (TF-IDF) values. In this study, TF-IDF values were used to identify changes in word use frequency over time. We computed Pearson correlation coefficients between each word’s TF-IDF trajectory and the proportion of messages that mentioned COVID-19–related words during the same text day. Only terms that fell below the false discovery rate threshold of 0.01 were selected. Analyses were conducted with packages that use the Python programming language. Spacy [15] and Textacy [16] were used for NLP analyses, and Scipy [13] and Statsmodels [17] were used for statistical analyses.

Since the selected words had no identifiable structure on their own, these words were assigned to empirically derived, human-validated topics by using Empath [18], which is a software program that assigns words to topical categories based on similarities in word use (ie, word embeddings). These categories were then validated by human curators. We calculated the percentage of words in each Empath-assigned topical category.

Empath category assignment is a nonarbitrary method for evaluating major topics that are associated with mentions of COVID-19, including positive and negative emotion states. However, this method does not categorize words based on diagnostic criteria. To determine the relationship between words that are associated with mentions of COVID-19 and words that are associated with diagnostic categories, words were converted into a dictionary, which was used to compare words with the publicly available International Classification of Diseases, Tenth Revision (ICD-10) symptom descriptions for the classification of mental and behavioral disorders [19]. Words and phrases that did not match existing diagnostic criteria were inspected for clinical relevance and reported as additional criteria.

All data were analyzed by using machine learning analytical methods. All data were deidentified prior to analysis. Patients who used the Talkspace service provided consent for using their aggregated and deidentified data for research purposes. Procedures for the collection of symptom questionnaires were approved by the Teachers College, Columbia University institutional review board (approval number: 15-426).

## Results

### Participants

We collected symptom data from 169,889 patients between January 1, 2017 and June 9, 2020. Most patients (88,444/160,807, 55%) were aged 26-35 years. Women accounted for 73.2% (124,358/167,559) of the included participants. A total of 60.3% (51,222/84,945) of participants identified as European American. There was a minimum of 2211 patients from every state. Most of the participants were from California (24,634/169,889, 14.5%) and New York (20,387/169,889, 12%). Furthermore, 44.6% (75,770/169,889) of participants reported that they were undergoing therapy for the first time (see Table 2).

**Table 2:** Demographic characteristics of the full sample (N=169,889).

Variable		Value, n (%)	Number of participants with missing data, n
**Age (years)**			9082
	18-25	38,594 (24)	
	26-35	88,444 (55)	
	36-49	23,960 (14.9)	
	≥50	9809 (6.1)	
**Education**			24,552
	Bachelor degree or higher	108,857 (74.9)	
	High school diploma	36,480 (25.1)	
**Race/ethnicity**			84,944
	European American	51,222 (60.3)	
	African American	14,271 (16.8)	
	Southeast/Asian American	8495 (10)	
	Native American	425 (0.5)	
	Other	10,533 (12.4)	
	Hispanic/Latinx	12,997 (15.3)	
**Gender**			2330
	Female	122,653 (73.2)	
	Male	40,382 (24.1)	
	Other	4524 (2.7)	
**Patients’ state of residence**			0
	California	24,634 (14.5)	
	New York	20,387 (12)	
	Texas	13,081 (7.7)	
	Florida	9174 (5.4)	
	Other US state	102,613 (60.4)	

### Outcomes

**Figure 1:**
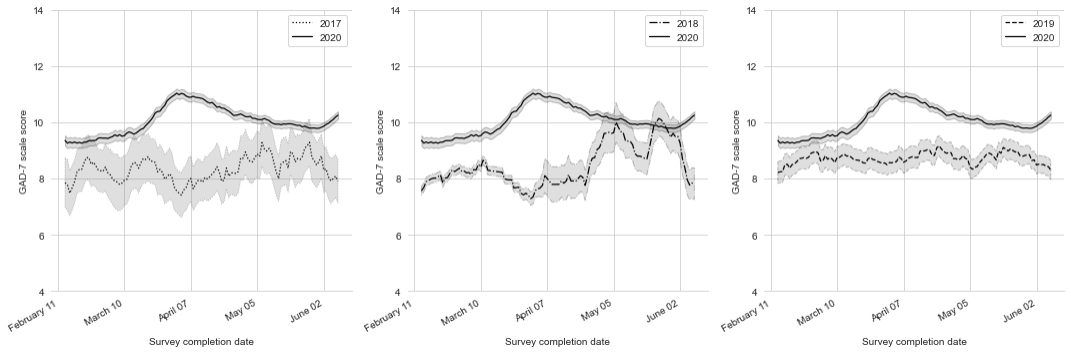
The 2-week GAD-7 score rolling averages from February 1, 2020 to June 9, 2020. The rolling averages from 2017, 2018, and 2019 are also presented. GAD-7: 7-item Generalized Anxiety Disorder questionnaire.

Based on the intake averages of the GAD-7 scores, a 1.42 increase (95% CI 1.18-1.65) in the average intake severity of anxiety symptom scores was observed in the 10,645 patients who underwent depression or anxiety treatment between March 15, 2020 and April 1, 2020. This was the period when GAD-7 scores were at their highest peak. As of June 9, 2020, there has been an ongoing 0.33 increase (95% CI 0.11-0.54; *P*<.001) in GAD-7 scores (see Figure 1). No significant changes were observed in 9-item Patient Health Questionnaire scores for intake depression severity (*P*=.79).

A total of 219,156 transcripts were identified and included in this study. These transcripts accounted for the 169,889 patients with available outcome data. The remaining 49,267 patients chose not to complete an intake assessment or had incomplete responses. All transcripts were analyzed, and 18.5% (40,448/219,156) of transcripts were found to contain mentions of COVID-19. Of the 500,000 words and phrases in the transcripts with mentions of COVID-19, 2377 (0.5%) positively correlated with terms that were associated with mentions of COVID-19, and 661 (0.1%) negatively correlated with terms that were associated with mentions of COVID-19. The words that correlated the most with COVID-19–related mentions were categorized as a set of complex reaction categories via Empath. These Empath categories included confusion and negative emotions (assigned words: 713/2377, 30%); health and medical emergencies (assigned words: 499/2377, 21%); work, business, and economic concerns (assigned words: 428/2377, 18%); technology and internet (assigned words: 285/2377, 12%); cleaning and hygiene (assigned words: 190/2377, 8%); government and leadership (assigned words: 166/2377, 7%); and traveling and shopping (assigned words: 96/2377, 4%). The Empath categories that negatively correlated with mentions of COVID-19 were party and celebration (assigned words: 198/661, 30%); positive emotion and love (assigned words: 179/661, 27%); friends and children (assigned words: 178/661, 27%); and optimism (assigned words: 106/661, 16%). Although the words that were associated with parties, celebrations, love, friends, and children were far less likely to co-occur with mentions of COVID-19, the word stem “lone” (eg, words like “alone,” “lonely,” “loneliness,” etc) was not considerably related to pandemic concerns.

The words and phrases in these Empath categories exhibited similarities to existing ICD-10 diagnostic classifications, including those for acute stress reactions (ICD-10 F43.0), which meets the criteria for trauma reactions (ICD-10 F43.1) if acute stress reactions persist over time; paranoia symptoms (ICD-10 F22); grief symptoms (ICD-10 Z63.4; symptoms need to persist for over 6 months to meet the full grief criteria); insomnia symptoms (ICD-10 G47.00); panic symptoms (ICD-10 F41.0); agoraphobia symptoms (ICD-10 F40.00); nonsuicidal self-injuries (ICD-10 Z91.5); obsession-compulsion symptoms (ICD-10 F42.9); and hypochondriasis symptoms (ICD-10 F45.21). Additional clinically relevant content included confusion about one’s state and difficulties in controlling anger at others and institutions (see Textbox 1).

**Descriptions of International Classification of Diseases, Tenth Revision diagnostic categories and symptoms that were identified via digital phenotyping.**

**Hypochondriasis (F45.21)**

- Worries of unexplained aches and pains (eg, head, back, joint, abdomen, and leg pain)
- Feeling that illnesses are not being taken seriously enough

**Insomnia (G47.00)**

- Problems with falling asleep, problems with staying asleep, and overall poor sleep quality

**Obsession-compulsion (F42.9)**

- Unpleasant thoughts, urges, or images that repeatedly enter the mind
- Feeling driven to perform certain behaviors or mental acts over and over again

**Paranoia (F22)**

- Feeling punished without cause
- Feeling sure one is being talked about
- Feeling that people are out to get you
- Feeling one must be on guard even with friends

**Grief (Z63.4)**

- Thoughts of a person who died make it hard do things one normally does
- Memories of a person who died are upsetting
- Feeling longing for the person who died
- Feeling angry about the death

**Acute stress (F43.0) and posttraumatic stress disorder (F43.1)**

- Experiencing an especially frightening, horrible, or traumatic event
- Having nightmares about the event(s) or thoughts about the event(s) when one did not want to
- Trying hard not to think about the event(s) or going out of the way to avoid situations that are reminders of the event(s)
- Feeling constantly on guard, watchful, or easily startled
- Feeling numb or detached from people, activities, or surroundings
- Feeling guilty or unable to stop blaming oneself or others for the event(s) or any problems the events may have caused

**Nonsuicidal self-injury (Z91.5)**

- Deliberately hurting oneself physically without intending to kill oneself or as a strategy for relief

**Panic (F41.0)**

- Experiencing panic episodes
- Worrying about having another episode

**Agoraphobia (F40.00)**

- Worrying about being in a public space in which escape might not be available should excessive anxiety or panic symptoms develop
- Obsessive, persistent, intense fear of open places

**Anxiety (F41.9)**

- Feeling afraid, as if something awful might happen
- Not being able to stop or control worry
- Worrying too much about different things
- Becoming easily annoyed or irritable

**COVID-19–specific psychological criteria**

- Feeling unsure about whether psychological reactions are normative or problematic
- Difficulty in controlling anger at others’ actions or lack of actions

## Discussion

In this study, we investigated the relationship between the COVID-19 pandemic and the intake anxiety and depression symptoms of treatment-seeking patients on a digital mental health platform. We identified a significant and noticeable increase in anxiety symptom severity, but not in depression symptom severity. We also applied machine learning methods to a large body of treatment transcripts via NLP methods, to identify additional symptoms that were associated with mentions of COVID-19, but would have been missed by symptom measures that assess anxiety or depression alone. These additional symptoms included those that were associated with other diagnostic categories, such as acute stress, posttraumatic stress disorder, grief, obsession-compulsion disorder, insomnia, hypochondriasis, nonsuicidal self-injury, and paranoia. In some ways, a more complex symptom profile can be generated via dimensional approaches to psychopathologic nosology [20,21], which focuses more on symptoms and functions rather than diagnostic categories. Our study suggests that tracking the lasting psychological impact of COVID-19 requires measures for a variety of symptoms from several disorders. To date, survey-based studies have accounted for a mix of depression, anxiety, insomnia, and stress-related conditions [22-24]. However, these studies have not reported data on the other symptoms that we identified in this study. Constructing an appropriate measure—whether by combining self-reported ratings with clinicians’ ratings or ratings from other sources, using the advantages of ecological momentary assessments, or developing other strategies for mitigating recall bias—is beyond the scope of this study. However, our study highlights that constructing appropriate measures is an important next step in applying our findings to practice.

An assessment that is composed of symptom questions that are informed by the appropriate measures could ultimately identify subpopulations of patients with different symptom profiles. This would assist with individualizing treatments and tracking heterogeneous responses to clinical interventions. For example, social isolation and loneliness are distinct risk pathways for suicide. Therefore, these risk pathways should be assessed as distinct behaviors, to inform treatment planning [25]. This is also the case for individuals with comorbid psychiatric disorders. Comorbidity is common, yet without pandemic-specific, longitudinal assessments, true comorbidity could be conflated with changing symptom constellations for the same underlying pathology. This has been exemplified in cases that demonstrate the dynamic interplay of bipolar disorder and anxiety symptoms [26]. Selecting symptom questions based on the rapid digital phenotyping methods that we implemented in this study can help reduce the burden on respondents, and provide a broader dimensional approach to monitoring psychopathology (ie, an approach that focuses on symptoms rather than diagnostic categories) [20]. Indeed, one of the goals of the National Institute of Mental Health has been to analyze disorders via a dimensional approach that does not rely on disorder categories, but instead draws on big data (ie, large clinical datasets) to increase our understanding of the underlying mechanisms of health and illness [27,28]. The data reported in this study are an important first step in the efforts for understanding symptom clusters that are associated with the pandemic, guiding the discovery of pathogenic mechanisms, and informing personalized interventions that maximize treatment benefits [29-31]. A critical next step for research is continuing to evaluate COVID-19 symptoms after vaccination and other programs begin to lower SARS-CoV-2 infection rates and reduce the threat of COVID-19. Commonly identified behaviors, such as social withdrawal, extreme anger, and COVID-19–related paranoia, may diminish after the pandemic and reflect adaptive responses to the pandemic. If, on the other hand, these symptoms persist, the possibility that other pathology mechanisms are at play increases, and further research would be warranted.

In our study, the lack of terms with the word stem “lone” may be in line with studies that have reported that loneliness is unlikely to be a major factor in COVID-19 pandemic–related psychological distress [24]. The lack of these terms also suggest that people are in fact isolated from others, given the few mentions of social topics in the text messages of patients with COVID-19–related complaints. The patients in this study may not yet think that isolation is similar to loneliness. The 18.5% (40,448/219,156) of patients who mentioned the pandemic exhibited a substantial increase in disease burden over and above that of the prestudy patient population that was already undergoing treatment. This finding corresponds with the increased number of new COVID-19 cases that was reported on the Talkspace platform. An important feature of this study is that we distinguish and quantify patients who seek care for COVID-19–related concerns, instead of patients who would have sought care without the influence of pandemic-related stressors. In this study, although anxiety symptom severity started to return to pre–COVID-19 pandemic levels between May 5, 2020 and May 30, 2020, we observed the opposite trend on early June 2020. It is thus advisable to continue focusing on patients who experience psychological symptoms and require treatment as a result of the pandemic.

Although this study offers a novel method and dataset for rapidly phenotyping COVID-19–related symptoms, it is not without limitations. First, our results may not be generalizable beyond the population of individuals who seek treatment through digital platforms. Second, our analyses relied on the longitudinal data of a convenience sample that self-reported their symptoms. We did not assess referral sources or use random assignment methods. Of particular note is the large number of women in our sample. However, this is consistent with existing data on the use of telemedicine services for routine care [32]. Despite these limitations, our results demonstrate the utility of large, unstructured data in rapid digital phenotyping methods for identifying psychological symptoms that are associated with patients’ COVID-19–related concerns, but are missed by standard depression and anxiety screening methods. The symptoms we identified in this study can be used to inform standard symptom surveys. Our study demonstrates how digital phenotyping can assist in and accelerate the development of traditional monitoring tools that do not require the use of digital therapy platforms or large amounts of textual material, avoid the potential for unwanted monitoring among technology users, and ensure that monitoring is an overt process (ie, people who are monitored are aware of being monitored).

## Abbreviations

GAD-7: 7-item Generalized Anxiety Disorder questionnaire
ICD-10: International Classification of Diseases, Tenth Revision
NLP: natural language processing
TF-IDF: term frequency-inverse document frequency
